# Antenatal care and opportunities for quality improvement of service provision in resource limited settings: A mixed methods study

**DOI:** 10.1371/journal.pone.0188279

**Published:** 2017-12-13

**Authors:** Andrea Solnes Miltenburg, Lisette van der Eem, Elias C. Nyanza, Sandra van Pelt, Pendo Ndaki, Namanya Basinda, Johanne Sundby

**Affiliations:** 1 Institute of Health and Society, Section for International Health, Faculty of Medicine, University of Oslo, Oslo, Norway; 2 Women Centered Care Project, a project of the African Woman Foundation, Magu District, Mwanza Region, Tanzania; 3 Department of Work and Social Psychology, Maastricht University, Maastricht, the Netherlands; 4 School of Public Health, Catholic University of Health and Allied Sciences, Mwanza, Tanzania; Public Library of Science, FRANCE

## Abstract

Antenatal care is essential to improve maternal and newborn health and wellbeing. The majority of pregnant women in Tanzania attend at least one visit. Since implementation of the focused antenatal care model, quality of care assessments have mostly focused on utilization and coverage of routine interventions for antenatal care. This study aims to assess the quality of antenatal care provision from a holistic perspective in a rural district in Tanzania. Structure, process and outcome components of quality are explored. This paper reports on data collected over several periods from 2012 to 2015 through facility audits of supplies and services, ANC observations and exit interviews with pregnant women. Additional qualitative methods were used such as interviews, focus group observations and participant observations. Findings indicate variable performance of routine ANC services, partly explained by insufficient resources. Poor performance was also observed for appropriate history taking, attention for client’s wellbeing, basic physical examination and adequate counseling and education. Achieving quality improvement for ANC requires increased attention for the process of care provision beyond coverage, including attention for response-based services, which should be assessed based on locally determined criteria.

## Introduction

Antenatal care (ANC) is an essential part of maternal healthcare services and includes history taking, screening for maternal illnesses such as hypertensive disorders and anemia, screening, prevention and management of infectious diseases, provision of prophylactic medication and essential health education [1–3]. Prior to the initiation of the Safe Motherhood Initiative, ANC was considered essential for identification and early management of high-risk pregnancies as a means to improve pregnancy outcomes [4], which later was argued to have little predictive value for reduction of mortality [5]. Global attention therefore shifted to emergency obstetric care and skilled care at birth as essential strategies to reduce maternal deaths [6,7]. Currently, however, it is globally accepted that while ANC alone is not *sufficient* to reduce morbidity and mortality, it remains an essential component in improving maternal and newborn health and wellbeing [8,9].

Considering the evidence of effectiveness of selected ANC services, a number of studies attempted to identify essential packages of interventions and required number of visits [5,10–12]. The aim to ensure access to goal oriented care for all women, and not only those at risk, resulted in the World Health Organization (WHO) recommendation of a minimum of four visits for low-risk pregnancies with targeted interventions in each visit [13], referred to as Focused ANC (FANC). FANC was meant to increase attention to the quality of ANC with emphasis on individual health education and counseling [3]. A reduction of the number of visits with targeted interventions in each visit proved to be equally effective as monthly ANC visits, although women were reported to be less satisfied with the new FANC model [5,14,15]. FANC was translated to settings in sub-Saharan Africa assuming equal benefits could be expected, despite the model not being tested in settings with low coverage of ANC visits and high mortality ratios [14]. Recently concerns have been raised that a reduced number of visits is associated with an increase in perinatal mortality, in particular in low-and middle-income settings [16]. Consequently the WHO updated their ANC guideline in 2016 aiming to provide women with a positive pregnancy experience and included a recommendation of a minimum of eight contacts [17].

In Tanzania, ANC coverage for the first visit has been above 90% since 1991, while coverage of four ANC visits has been stable around 60% over the past decades [18]. The Tanzanian Ministry of Health, Community Development, Gender, Elderly and Children (MoH) introduced FANC at the national level in 2002, changed policy guidelines for ANC, adapted documentation requirements such as administering new ANC cards and provided training to health care workers (HCW’s) [19]. Initial indicators developed to assess ANC focused on timing and number of visits and more recent indicators have increasingly included assessment of the quality of the content of ANC [20]. Since introduction of FANC, several studies in Tanzania have assessed the quality of ANC and reported poor adherence to FANC guidelines and insufficient coverage of routine practices, in particular for health education and counseling [21–27].

Quality of care assessment is complex and requires attention to health system components such as available human resources, supplies and infrastructure (*structure*), the process of provision of routine health services and timely action in case of complications which is based on evidence-based practices (*process*), and coverage of key practices, health outcomes and satisfaction of both provider and client (*outcome*) [28–30]. Additional considerations for assessing quality include the extent to which care provision is people-centered with a focus on respect for patient dignity, while minimizing unnecessary interventions and harmful practices as well as efficiency in minimizing wastage and maximizing resource use [29,31].

The majority of previous research studies have focused their quality assessment of ANC primarily on coverage of a number of routine ANC practices [20,21,25–27,32,33] or the experience of care [34–37]. Determinants of poor quality of ANC are poorly understood and remain limited to lack of (human) resources and poor health worker knowledge [22,25–27,34,35,38]. Care provision, however, is more than deliverance of a number of health interventions and includes complex care process such as prioritization of services, interpretation of findings and clinical decision-making [28]. Additionally, quality is influenced by the complex context in which services are provided, in particular in low-resource setting. This study aims to assess the quality of antenatal care provision from a holistic perspective in a rural district in Tanzania. First we explore the organization and structure of the care setting. Second, the process of care provision is assessed related to evidence-based guidelines. Thirdly we examine the coverage of essential practices and women´s experience of care. Finally, we reflect on areas of suboptimal care and discuss opportunities for quality improvement.

## Materials and methods

### Study setting

This study took place in Magu district, in the region of Mwanza, Tanzania, which borders Lake Victoria, as part of an exploratory and baseline study for the Woman Centered Care Project (WCCP), which is a project of the African Woman Foundation. For further information we refer to prior publications [39–41] and their website (www.africanwomanfoundation.com). Magu district has a population of 299,759, with 23% women of childbearing age [42]. It has 31 government health facilities that provide ANC services including a district hospital, four health centers and 26 dispensaries. The district hospital is approximately 65km from the city of Mwanza where there is a regional referral hospital and a consultant hospital. The ANC attendance rate for the first visit has been above 95% every year since 2011. The proportion of births delivered in health facilities was estimated to be 65% in 2013 [43].

### Study design

This paper draws upon data collected over several periods from 2012 to 2015 making use of multiple data collection methods. Quantitative methods included: facility audits of supplies and services, ANC observations and exit interviews with pregnant women. Qualitative methods included participant observation, semi-structure interviews and focus group discussions.

#### Quantitative data collection

For the facility audits a survey tool was developed based on the Johns Hopkins Program for International Education in Gynaecology and Obstetrics (JHPIEGO) manual for monitoring birth preparedness and complication readiness to assess facility readiness to deliver maternal health services [44]. For the ANC observations a checklist was used based on Focused ANC guidelines by Kearns et all (2014), the Maternal and Newborn Quality of Care Survey ANC Observation Checklist developed by the Maternal and Child Health Integrated Program (MCHIP) and the currently used ANC card from MoH in Tanzania [45,46]. Tools were pre-tested in a health facility outside of the research area. Based on experience from 2014 minor improvements were made for assessment in 2015. Exit interviews included review of documentation of services on the ANC card, assessment of knowledge related to birth preparedness and complication readiness including danger signs and evaluation of satisfaction of care. Exit interviews were held at the health facility.

ASM and SP visited health facilities in April-May 2014 and 2015 for the facility audit together with representatives of the district medical officer to collect information on facility infrastructure, available services, equipment and supplies. In 2014 the audit was conducted while facilities were awaiting their new batch of quarterly supplies. In 2015 the audit was done two weeks after receiving new supplies. Facility statistics were collected using the Health Management Information System registries (HMIS) of the year 2013 and 2014. In August-September 2014 and 2015 Tanzanian medical students from the Catholic University of Health and Allied Sciences, School of Public Health (CUHAS-SPH) in Mwanza conducted ANC observations and exit interviews and were trained and supervised by EN, NB and ASM in 2014 and PN, SP and LE in 2015.

Purposive sampling was used to select health facilities and villages in 9 of 18 wards of the district, based on population size (three large populated, three middle populated and three small populated areas) and inclusion of different geographical locations in the district covering both areas close to the lake as well as more in-land and close to the main tarmac road. Health facilities included 13 dispensaries, one health centre and one district hospital. For ANC observations a sample size was calculated to be 422 with OpenEpi, version 3, open source calculator with a confidence interval of 95% including an expected refusal rate of 10%. Health facilities were visited on a minimum of two different occasions until the sample size was reached to ensure observations at different clinic days during the week. All women visiting the health facilities for ANC during these days were eligible for inclusion. Due to logistical reasons the sample was not reached in 2014. In 2015 After the ANC observations all women were approached for exit interviews and 286 (69%) agreed to participate. Main reason for refusal was lack of time.

#### Qualitative data collection

ASM and LE (both medical doctors) and SP (nurse) performed regular participant observations in the selected health facilities during the entire study period and had numerous informal conversations with health workers, district leaders, pregnant women and their husbands. ASM and SP have lived in Tanzania for more than two years and had most conversations in Kiswahili. A translator assisted LE. Masters students from medicine and public health programs in the Netherlands (VU University Amsterdam and University of Utrecht) conducted interviews and focus group discussions supervised by ASM. Students had their own sub-topics of interest but together covered aspects related to availability, accessibility, acceptability and quality of maternity care, including care during pregnancy and birth.

Participants for interviews were selected through convenience sampling and included women of reproductive age, pregnant women and women who recently gave birth (N = 48), men including partners of pregnant women (N = 31) and health care workers (N = 26). On average 8 participants participated in each focus group and were held with women (N = 2), men (N = 1), mixed gender (N = 2) and with health care workers (N = 7). Participants were recruited with help of the village executive officers and health workers in the study area or during clinic hours at the ANC clinics. Interviews took place at the house of the participant and focus group discussions at a convenient location for all participants.

### Data analysis

Quantitative data was entered using the Magpi mobile data collection platform [47], which uses mobile phones, allowing for digital data entry during data collection and reducing entry errors. Data from the Magpi data collection software were exported to Excel (version 14.5.7 and SPSS (version 22) for analysis. Overview of supplies and materials across facilities were made in Excel. For ANC observations and exit interviews descriptive analysis was performed using SPSS. Facility audit and ANC observations were performed both in 2014 and 2015. For this paper data of both years were analysed separately. We did not perform a comparative analysis, as this was not the objective of this paper.

Qualitative data included field notes and reflective journals as well as transcripts from interviews and FGDs. Interviews were mainly held with a translator and were transcribed in English. For some interviews also Kiswahili was transcribed and translated by research assistants to verify the quality of the translation. Data was initially analyzed using different frameworks depending on the main aim of the sub-studies. For the purpose of this paper qualitative findings was re-analyzed using a quality of care framework as adapted from the WHO vision of quality of care for pregnant woman and newborns (29). Qualitative data was used to provide context to the quantitative findings and assisted to increase understanding and interpretation of the complex care processes, in particular where findings deviated from evidence-based guidelines. Triangulation of data was done through a continuous back and forth interpretation of findings. When new information emerged along the way this resulted in adaptation of tools, development of new hypotheses and alternative explanations, which were then, further assessed and analyzed.

#### Validation of data

In addition to the project team’s extensive field engagement and triangulation of multiple sources of data, member checks and peer debriefing during the data collection period assisted in interpretation and validation of our findings. The Magu District Council and Ward District Councils received reports of the plans and outcomes of all research activities since 2012 through frequent feedback meetings. Some of these meetings included facility based and community based health workers. Feedback meetings were formalized in the form of quarterly meetings in 2014. Finally, the ANC observations were discussed during regular meetings with the authors and extra observations in some of the facilities for counter check of the data collected and to gain further understanding of the local setting. Preliminary results of the 2015 ANC observations were discussed and reflected upon with health care workers in all thirteen facilities to further understand findings.

### Ethical considerations

Ethical approval for this study was obtained from the National Institute for Medical Research (MR/53/100/103) and the VU University Amsterdam in the Netherlands (2013/135). The Regional Committee for Medical and Health Research Ethics, Section A, South East Norway (2015/1827), and the Norwegian Social Science Data Service (44482/3/MHM) both reviewed the study. Research clearance was acquired from the Tanzania Commission For Science and Technology. Written informed consent was obtained from health workers and verbal informed consent was obtained from pregnant woman prior to ANC observations and exit-interviews. Participants of interviews and FGDs were asked for verbal consent. Health Care Workers and pregnant women were explained on the purpose of the study and that they were free to withdrawal their participation once enrolled without affecting their health care services at the specific health facility.

## Results

### Organization of care

ANC is provided daily at the district hospital and health centers. Most dispensaries have daily ANC clinics lasting officially from 8am to 2pm. Specific days are often allocated for women coming for their first visit. In the morning nurses provide group education, usually on a different topic every month including prevention of malaria, HIV/AIDS, birth preparedness and breastfeeding. This is followed by registration of each woman. In the larger facilities this is usually done in larger groups together with weight measurement in the waiting area of the clinic. At the district hospital women in need of investigations are sent to the different locations (for HIV testing or to the laboratory for other blood tests). HIV testing does not always seem optional as women are not always asked for consent and privacy is not guaranteed depending on the facility infrastructure and patient flow. Women who come without their husbands have to wait, couples have priority. Sometimes women are refused care for their first visit if they don’t bring their husband [39]. For the physical check-up women are seen in private rooms. Depending on the type of visits and location, women can spend between 5 minutes up to an entire day at the facility. ANC observations done in 2015 revealed both first and return visits had a mean duration of 11 minutes. When women inform nurses they are ‘sick’, have symptoms or findings that deviate from “normal”, they are sent to the clinical officer or clinical attendant if present at the facility. At health centers and dispensaries similar patterns of care provision are seen. Some however provide ANC for all women, one by one, where women are seen alone in private rooms for all services needed during that visit.

### Documentation

The ANC card, which is provided by the government, is meant to assist health workers in the provision of FANC and is kept by women themselves. Health workers can refer to the MoH ‘learners guide for ANC service providers’ for guidance on ANC. Health workers have two additional government ANC registration books that remain at the health facility. Each book needs to be filled in after each visit including an attendance registry with socio-demographics and registry for documentation of services provided for each woman. There are additional separate books for HIV testing and treatment if women are HIV positive.

The ANC card has three parts allocated for documentation of services during pregnancy, birth and after birth. In practice the card is mostly used for ANC and documentation of the outcome of this pregnancy. Three categories of referral indication distinguish between risks needing referral for further assessment during pregnancy, referral for birth or immediate referral. The main part of the card, which is filled in during each visit, includes essential services that need to be provided based on the FANC guidelines. It is recommended women with uncomplicated pregnancies receive a minimum of four visits. Women with complications need appropriate referral or additional visits depending on individual conditions, it is however not specified which actions are expected for which conditions. At the same time there is no space on the ANC card for health workers to document findings other than what is offered on the card (for example, if women express any complaints or symptoms, if findings deviate from normal, second opinions by colleagues, documentation of decision making, treatment provided by the health worker or advice for referral for additional services). There is also no space to document admissions or additional services received during a pregnancy. If services are not being provided due to lack of supplies the MoH guideline advises that ‘the provider should encourage the client to return when it is expected that the supplies will be available’ [19]. In practice, however, health workers explained that the availability of such supplies is highly variable and therefore women are usually requested to come back the following month. If the ANC card is out of stock women report they are encouraged to buy their own notebooks that will then be used instead, often they remain with these notebooks until birth, despite availability of the ANC card during next visits.

### Facility appearance and resources

Table 1 presents the results of the Facility Audit in 2014 and 2015. Conditions of the facilities varied. Some facilities were newly renovated, mostly located close to the main tarmac road; others were in state of collapse and unclean with molding walls, holes in the roofs and windows and bird nests inside the facility. Water source and electricity was not available in all facilities. Magu District has been dealing with major shortages of water, despite its location bordering Lake Victoria. Most facilities get water from the closest well or need to purchase it. In some cases women are told to bring water to the facility. The issues of cleanliness and availability of electricity and water as well as lack of toilets or health worker housing is a major concern for health workers, community members and community leaders and a frequent discussed topic during community group meetings.

**Table 1:** Facility audit outcomes and availability of supplies for antenatal care provision.

Audit elements		District Hospital 2014 (N = 1)	Health centre 2014 (N = 1)	Dispensaries 2014 (N = 13) N (%)	Dispensaries 2015 (N = 13) N (%)
**ANC equipment**	Stethoscope	Yes	Yes	10 (77)	9 (69)
	BP machine	Yes	Yes	8 (62)	9 (69)
	Fetoscope	Yes	Yes	13 (100)	13 (100)
	Thermometer	Yes	Yes	9 (69)	9 (69)
	Urine dipstick	Yes	None	1 (7.7)	1 (7.7)
	Tape measure	Yes	Yes	11 (85)	13 (100)
**Medication ANC**	Iron/Folate	Yes	None	12 (92)	12 (92)
	Anti-Malaria	Yes	None	4 (31)	7 (54)
	Tetanus Toxoid	Yes	None	11 (85)	12 (92)
	Paracetamol	Yes	None	5 (38)	12 (92)
	ARVs	Yes	None	8 962)	13 (100)
**Blood tests**	Hb	Yes	Yes	0 (0)	0 (0)
	Malaria	Yes	Yes	4 (31)	13 (100)
	HIV	Yes	Yes	2 (15)	13 (100)
	Syphilis	Yes	Yes	0 (0)	8 (62)
	Blood grouping	Yes	No	0 (0)	0 (0)

The number of ANC visits varied between dispensaries ranging from 9–49 new first ANC visits per month. During the audits health workers explained that the two dispensaries with more than 30–50 new first ANC visits per month receive many women from outside of their catchment area, who come from neighboring districts and surrounding wards that have fewer health facilities. Their staffing levels, however did not differ from less populated facilities. Dispensaries had between 2–4 staff members, mostly consisting of one medical attendant, one nurse/midwife and a clinical officer. Some facilities have a number of nursing students present who are performing much of the work. Three dispensaries actively work with local traditional birth attendants who both see women for ANC and assist births at the facilities.

None of the facilities had all equipment and medication needed for provision of ANC. In 2014 only one dispensary was able to both measure blood pressure and check for protein in the urine. However, during observations it appeared the staff did not use the urine dipsticks because they were unaware how to use them. None of the dispensaries in 2014 or 2015 were able to test hemoglobin level, blood grouping or test for syphilis and in 2014 few facilities had the ability to test for Malaria or HIV. While reviewing the facility statistics it appeared every year there were certain periods in the year where HIV tests were done frequently. During facility visits staff members commented that there is a frequent stock-out of HIV tests and stock is often only available for a few weeks per quarter.

### ANC care provision

Results of the ANC observations are presented in Table 2. Of all ANC observations conducted, the majority of observed ANC consultations were with women present for their 2^nd^ or 3^rd^ visit. In 2014 and 2015 respectively 16% percent and 20% of the women were pregnant for the first time. In 2014 20% of the observations took place in the district hospital, whereas in 2015 this was 10%. In 2015 a skilled health provider provided the majority of ANC services. Medical attendants provided services during 29% of the observed visits, sometimes under supervision of a clinical officer.

**Table 2:** Services provided during ANC consultations (Process).

Year		2014 (N = 250) N (%)	2015 (N = 414) N (%)
**Visits**	1^st^ visit	97 (39)	115 (28)
	2^nd^ - 3^rd^ visit	125 (50)	243 (59)
	≥ 4 visits	28 (11)	56 (13)
**History (1**^**st**^ **visits)**	Medical history	65 (67)	37 (32)
	Obstetric history (para ≥ 1)	57 (69)	83 (97)
**Examination (all visits)**	Weight	241 (96)	408 (99)
	Pallor	150 (60)	81 (20)
	Oedema	196 (83)	87 (21)
	Blood pressure	129 (52)	116 (28)
	Fetal presentation (visit ≥ 3)	68 (85)	122 (83)
	Fundal Height	231 (92)	394 (95)
	Fetal heart rate (visit ≥ 2)	138 (90)	287 (96)
**Laboratory tests (1**^**st**^ **visit)**	Haemoglobin	13 (13)	2 (1.7)
	HIV	32 (33)	106 (92)
	Syphilis	1 (1)	0
	Urine test	0	1 (1)
	Grouping/Rhesus	0	0
**Medication**	Ferrous sulphate / folic acid (all visits)	215 (86)	307 (74)
	Mebendazol (1^st^ visit)	-	36 (31)
	Sulphadoxine-pyrimethamine (2^nd^/3^th^ visit)	35 (28)	220 (91)
	Tetanus toxoid (1^st^/2^nd^ visit)	104 (61)	168 (63)
**Education (all visits except where indicated)**	Birth preparedness and complication readiness	198 (79)	282 (68)
	Danger signs in pregnancy	189 (76)	227 (55)
	Family planning (visit ≥ 3)	27 (34)	60 (41)
	Effect of STI/HIV/AIDS	122 (49)	155 (37)
	Diet and nutrition	144 (58)	60 (15)
	Use of drugs in pregnancy	116 (46)	71 (17)
	Process of pregnancy	199 (80)	38 (9)
	Personal hygiene (1^st^ visit)	59 (61)	10 (9)
	Rest and exercise in pregnancy	150 (60)	10 (2)
	Symptoms/signs of labour (visit ≥ 3)	52 (65)	9 (6)
	Harmful habits (e.g. smoking, drug abuse)	54 (22)	1 (0)
	Return visit	208 (83)	60 (15)
**High risk (all visits)**	High risk pregnancy identified	-	77 (19)
	Referral	7 (3)	36 (9)

History taking for first visits was assessed differently in both years after adapting our observation tool to be more specific. Where in 2014 the majority of health workers asked *some* medical and obstetric history, in 2015 it became clear that history taking rarely included risk identification based on possible problems during previous pregnancy (see Table 3). Additionally few women were asked if they had any current complaints or problems (20%), were asked about the presence of danger signs (6%) or asked about the presence of fetal movements (3%). Interviews with pregnant women revealed women rarely mention if they have problems without being asked for it and they assume that if health workers don’t report problems, their pregnancy is progressing well. In 2015 28% of the women were informed about their expected date of birth, and 55% was informed about their gestational age, usually in months of pregnancy. The gestational age per visit is mostly based on the documented gestational age during the previous visit or based on and equal to centimeters of the fundal height. These estimations do not always match the last normal menstruation period or the expectations of the women causing confusion about the estimated date or month of birth.

**Table 3:** Specific assessment of history taking in 2015.

History	Specific assessment	N (%)
**Medical history (1**^**st**^ **visit, N = 115)**	HIV status	107 (93)
	History of TB/heart disease/diabetes	34 (30)
	Use of medication	21 (18)
**Obstetric history (para ≥ 1, N = 86)**	Gravidity/Parity/Living children	83 (97)
	Mode of previous birth	10 (12)
	Previous PPH	10 (12)
	Previous high BP in pregnancy	1 (1)
**Current pregnancy (all visits, N = 414)**	Asked about any complaints	83 (20)
	Asked about any danger sign	25 (6)
	Asked about fetal movements	13 (3)

Clinical examination varied between 2014 and 2015. In both years however during almost all visits women were checked for their weight, fundal height and fetal heart rate. In 2014 blood pressure was measured in 53,4% while in 2015 this was 28%. At the district hospital, blood pressure was measured in only in 2 out of 42 observations. Some facilities that had a well-functioning blood pressure machine did not always use them during observations: to illustrate, in 2015, out of 313 observations done, the blood pressure machine was only used 37.1% of the time. This was largely attributed to the fact that not all medical attendants were aware how to use the machine; other health workers reported malfunctioning of the machines. Provision of medication and laboratory investigations corresponded with the availability or lack of supplies.

Few women were identified as high risk and referred for ANC at higher level. Women with high parity or age below 20 were mostly advised to give birth at the hospital or health workers documented this specifically on the ANC card. Abnormal findings, if any, were rarely a reason for referral and return date was usually standardly documented to be the next month, independent of number of visits, current gestational age, complaints or other factors. Also women, whose gestational age was estimated to be above 36 weeks, could be requested to come back the next month, sometimes with a return date *after* the estimated date of birth.

### Experience of care

Exit-interviews were done with 286 (69%) women whose visits were observed. Knowledge of birth preparedness and complication readiness including danger signs is presented in Table 4 and was similar to those topics which were primarily discussed during the ANC visit. Thirty eight percent of women interviewed stated that they did not receive any explanation as to why certain examinations and procedures were performed, and 27% of women were never asked if they had any questions about their health status. Despite this, 96% of women interviewed said they were ‘happy with the care received’ and 97% would recommend this facility to family and friends. Furthermore, 92% of women interviewed planned to give birth at the facility where the consultation was observed by the project team. In contrast to these positive statements in the exit interviews, during community group meetings and focus group discussions, community members expressed frequent concerns regarding long waiting times, unavailability of staff, lack of essential medicines and equipment as well as poor health worker attitudes both towards women and their partners.

**Table 4:** Birth preparedness and danger signs.

Topic	Items	ANC Observations 414 N (%)	Exit interviews with women 286 N (%)
**Birth preparedness**	Purchase relevant items	259 (63)	135 (47)
	Financial arrangements	227 (55)	67 (23)
	What to do for a complication	196 (47)	3 (1)
	Transportation	139 (34)	11 (4)
	Place of birth	43 (10)	25 (9)
	Skilled birth attendant	30 (7)	1 (0)
**Danger signs**	Vaginal bleeding	220 (53)	71 (25)
	Severe headache or blurred vision	202 (49)	35 (12)
	Swelling of fingers, face, legs	190 (46)	22 (8)
	Severe abdominal pain	183 (44)	32 (11)
	Fever or too weak to get out of bed	95 (23)	16 (6)
	Convulsions	37 (9)	9 (3)
	Fast and difficult breathing	14 (3)	5 (2)

## Discussion

This study aimed to assess the quality of antenatal care provision from a holistic perspective. Similar to studies in other regions of Tanzania, major resource problems regarding both the availability of sufficient qualified staff as well as materials needed for ANC provision mitigate poor delivery of adequate care [22,25,38]. Frequent occurrences of out-of-stock medication and materials even after receiving a new batch of quarterly supplies indicate serious supply chain problems, which are not easily solved at facility level. However, quality of care requires more than adequate resources and our findings indicate that it is unlikely that an increase in resources alone will improve the quality of ANC. Availability of supplies did not necessarily mean they would be used nor did the presence of a skilled health worker ensure the provision of essential ANC services. On the contrary, this study revealed that many routine ANC services were neglected while priorities for other services seemed to be more out of habit than instrumental for clinical reasoning and decision-making.

Similar to previous studies our findings indicate weaknesses in the provision of appropriate history taking, attention for danger signs and provision of essential health education as well insufficient screening for anemia, hypertension, malaria and syphilis [21,22,25,26,48]. Although the ANC card functions as a ´working guide´ [38] not all services indicated on the card are always provided and health workers seem to prioritize some services over others. Measurement of weight, fundal height and fetal heart rate are nearly always done while other services are frequently left out, partly dependent on availability of supplies. Over the years, the provision of ANC has turned into a complex ‘mosaic’ of services [6] with specific attention for integration of vertical interventions proven to be effective, such as provision of tetanus toxoid, iron and folic acid supplementation and voluntary counseling and testing for HIV [3, 49]. Struggling to implement the increasing number of interventions during busy ANC clinics and limited time available, health workers seem to cope by prioritizing services either because they are easily provided or for which they believe they will be held accountable [38,49]. Prioritization can also be influenced by client expectations, who may report high satisfaction, despite poor quality of services, as long as tangible services such as medication and vaccinations are provided [26,35]. Most of the clients in this setting have never experienced excellent care, so their expectations may not be what we should attempt to measure.

Provision of ANC requires many different actions and skills; next to routine services it requires provision of ´response based services´ including: responding to clients symptoms and concerns; clinical reasoning and skills; analytical assessment of findings; adequate decision-making; risk identification; proper client counseling [6,13]. This means services, which are tailored to the individual woman’s needs, to ensure a positive pregnancy experience [17]. Coverage of routine services alone is insufficient if information is not used or acted upon. Some routine services such as weight and fundal height measurement have little meaning for progress of pregnancy if they are not related to the gestational age and progress over time [50]. Similarly, it is questionable if the health worker is fully informed of maternal and fetal wellbeing if women were not asked about fetal movements, presence of danger signs or given opportunity to express complaints and ask questions. Response based service provision also means services need to be adapted to the local context. When no blood pressure machine or urine dipstick is available it is easy to assume that women presenting with risk of pre-eclampsia no longer can be identified [51]. However proper history taking, assessment of danger signs and targeted physical examination including assessment of oedema and fetal wellbeing are equally important, especially in low resource settings. In the absence of resources, performing such basic assessments and responding adequately, is the highest attainable standard that can be expected and therefore should be considered of ‘good enough’ quality.

Health policies and evidence-based guidelines shape service provision, and evaluations relate clearly to those. Current guidelines and quality indicators for ANC are limited to a number of routine services. Quality assessments based on these indicators however do not provide sufficient information whether women received the right services based on their individual needs. Assessments are insufficiently approached along the continuum from antenatal to delivery care, while early identification of danger signs during ANC has the potential to reduce the need to resort to costly and dangerous interventions during delivery [52]. Additionally, aiming for quality improvements without attention for the process and local dynamic circumstances in which they are delivered is likely to fail [49,51,53]. Output indicators should therefore not be limited to coverage of some routine services alone and include indicators regarding client wellbeing as well as responses to findings and basic assessments such as history taking and physical examination. Ultimately these indicators should also be linked to specified, well-known fetal and maternal outcomes. Training and tools available to health workers, such as the ANC card, may not be adequately adapted to the provision of quality ANC in their working environment [54–57]. Therefore, changing quality indicators requires extensive training and supportive supervisions to assist health workers how to cope within their dynamic local settings. This requires increased attention for basic midwifery skills including clinical competence and problem solving, rather than focus on the routine indicator-documentation alone. Broadening the quality indicators and adapting guidelines, reporting and supervision for quality improvements based on local realities allows for incremental improvement of quality [57]. The expansion of the new ANC model to include eight contacts will be a major future challenge for improved quality [17]. Local practice needs to be carefully adjusted to allow for a gradual change, and also cater for those women that still only go 1–4 times.

Limitations of this study prevent making generalized conclusions, but nevertheless the context in terms of what was observed in facilities is representative of the situation for semi-rural areas in Sub-Saharan Africa. Although the facility audit was done twice and always together with a government representative it is possible that availability of supplies was not adequately reported. The audit and observations were done in different periods in the year and several data checks aimed to increase validity of both the audit and ANC observations which helped to fine-tune irregularities. Differences between 2014 and 2015 observations can be explained by a difference in supply levels or changes in staffing. However, it is likely that inter-observer variability caused this difference. Based on the 2014 experience we specified the observation tool for 2015 to increase our understanding. Nevertheless, in particular for education and counseling, inter-observer differences can have occurred and illustrates the challenges with such quality assessments. There are no criteria available to determine when sufficient education is provided on a given topic for each individual woman. We relied solely on qualitative methods to gain an insight into the quality of response-based services. Direct ANC observations did not give us information whether women received the appropriate services as observers could not check or see the outcomes of the findings nor how the health worker interpreted these. Further research is needed to understand these processes. Despite these limitations the combination of different research methods as well as the qualitative elements of the data collection process shows to be relevant, similar to other comparable studies in this field [34,38,56].

## Conclusion

Inadequate resources contribute to poor quality of ANC and many routine ANC services seem to be neglected. Nevertheless, coverage of routine services alone is not sufficient for provision of quality ANC services and there should be increased attention for the importance of basic assessments and response based services. Current quality indicators and ANC guidelines do not provide sufficient guidance for achieving quality ANC adapted to the individual woman’s needs. Flexible quality assessment criteria adapted to the local setting should be considered to strive for realistic quality improvement of ANC.
